# Ultrasound‐Responsive Microcapsules Delivering Oxygen and Traditional Chinese Medicine for Wound Healing

**DOI:** 10.1002/smmd.70021

**Published:** 2025-11-16

**Authors:** Baojie Wen, Danqing Huang, Chuanhui Song, Yi Chen, Yuanjin Zhao

**Affiliations:** ^1^ Department of Ultrasound Institute of Translational Medicine Nanjing Drum Tower Hospital Affiliated Hospital of Medicine School Nanjing University Nanjing China; ^2^ State Key Laboratory of Bioelectronics School of Biological Science and Medical Engineering Southeast University Nanjing China

**Keywords:** core‐shell microcapsule, curcumin, drug delivery, oxygen carrying, sonodynamic antibacterial therapy

## Abstract

Hydrogel microparticles with different actives encapsulation have reliable efficacy in wound repair, and the challenge is to improve the active substance to improve the efficacy. Here, a novel ultrasonic responsive core‐shell microcapsule delivery system is proposed, which can simultaneously load traditional Chinese medicine sonosensitizer and oxygen synergistic effect to promote the healing of infected wounds. The microcapsule has a core of oxygen‐rich perfluorocarbon (PO) and a hydrogel shell with curcumin (PO/C‐MC). Ultrasound can trigger the release of oxygen from perfluorocarbon and stimulate the sonodynamic effect of curcumin, which is not only an anti‐inflammatory traditional Chinese medicine but also a sonosensitizer, thus realizing the synergistic treatment of traditional Chinese medicine and acoustic dynamics. Under the irradiation of low‐intensity ultrasound, PO/C‐MCs can effectively increase the oxygen concentration and enhance the antibacterial effect of sonodynamic therapy, synergizing with the anti‐inflammatory effect of traditional Chinese medicine to promote the rapid healing of chronic infected wounds. The study demonstrated that ultrasound‐responsive PO/C‐MCs microcapsules significantly enhanced the healing process of infected wounds in mice. Given these beneficial characteristics, PO/C‐MCs represent a promising therapeutic candidate with considerable potential for clinical application in managing chronic infected wounds.

## Summary

- Core‐shell microcapsules with ultrasonic response characteristics were successfully prepared by microfluidic technology, which have excellent oxygen‐carrying capacity and drug‐release capacity.
- An ultrasound‐responsive core‐shell microcapsule drug delivery system capable of simultaneously loading oxygen and Chinese medicine sonosensitizer has been developed for the treatment of infected wounds.
- In an infected wound mouse model, the ultrasound‐responsive oxygen‐carrying and curcumin microcapsule delivery system demonstrated antibacterial and anti‐inflammatory effects and promoted the rapid healing of infected wounds.

## Graphical abstract

An ultrasound‐responsive microcapsule simultaneously loading oxygen and Chinese medicine sonosensitizer has been developed for sonodynamic therapy against bacterial infection. In an infected wound mouse model, this microcapsule delivery system demonstrated antibacterial and anti‐inflammatory effects and promoted rapid healing of infected wounds.

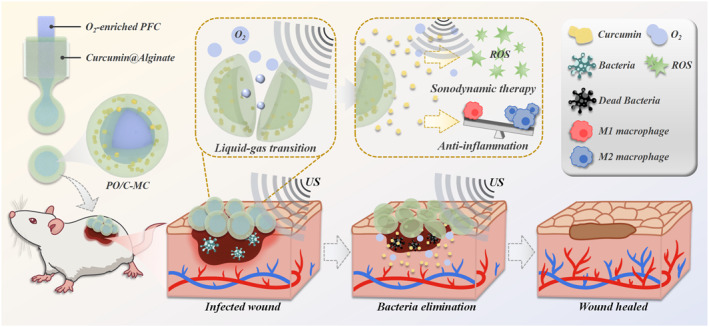

## 1 Introduction

Wound treatment is a very common problem in clinical practice and deserves attention. The wound healing process might be affected by many factors, among which inflammation, bacterial infection, and hypoxia are the most important [1]. In terms of alleviating inflammation, numerous traditional Chinese medicines show excellent effects, such as curcumin, berberine and emodin etc [2, 3]. These traditional Chinese medicines can inhibit the production and release of inflammatory mediators and interfere with binding to receptors [4, 5]. Notably, recent studies have found some traditional Chinese medicines featured with ultrasound responsiveness and can serve as sonosensitizers, showing great potential in facilitating sonodynamic therapy against bacteria [6, 7]. On the other hand, in order to reverse the hypoxic state of wounds, the current methods are mainly focused on improving wound dressing's permeability or applying hyperbaric oxygen therapy [8, 9]. Although some progress has been made, these oxygen supplies often require complex equipment and lack controllability. Additionally, considering the multiple factors affecting wound healing, multiple administrations and dosages are inevitably required, increasing the challenge and difficulty of chronic wound treatment [10, 11]. Therefore, new therapeutic strategies remain to be developed to realize controllable anti‐inflammatory drugs and oxygen supply in promoting wound treatment.

**FIGURE 1:**
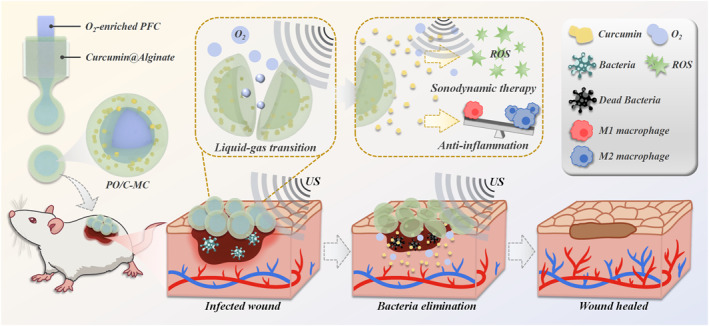
Schematic diagram of PO/C‐MCs + US system for the treatment of an infected wound mouse model. The ultrasound‐responsive microcapsule delivery system can deliver oxygen and herbs simultaneously, promoting the healing of infected wounds through sonodynamic antibacterial effects and Chinese herbal anti‐inflammatory mechanisms.

In this study, we propose an ultrasound‐responsive drug microcarrier via microfluidic technology to co‐deliver oxygen and traditional Chinese medicine for wound treatment, as shown in Figure 1. Microcarriers have been widely applied in biomedical fields, especially drug delivery [12, 13]. To obtain microcarriers with desired functions, various technologies have been developed [14, 15]. Among them, microfluidics is a technique for the precise manipulation of fluids in micron‐ and nano‐scale, which allows the preparation of microcarriers with finely tuned architectures [16, 17]. By using materials with different properties as components of microcarriers, the co‐delivery of multiple active substances can be achieved [18, 19]. Noteworthy, based on the characteristics of the microcarriers and microcargoes, the corresponding microcarrier structure can be designed according to the maneuverable microfluidic channels to achieve controllable drug activation and release [20, 21]. Therefore, it is anticipated to apply microfluidic technology to construct a platform for controllable oxygen and traditional Chinese medicine delivery to promote wound healing.

Here, we presented the desired ultrasound‐responsive oxygen and curcumin co‐loaded microcapsules fabricated by microfluidics for the treatment of infected wounds. Based on a capillary microfluidic device, we constructed microcapsules with oxygen‐enriched perfluorocarbon (PO) core and curcumin‐encapsulated hydrogel shell (PO/C‐MCs). As both an anti‐inflammatory drug and sonosensitizer, curcumin can be released sustainably from the shell. Under the stimulation of low‐intensity ultrasound (US), the liquid perfluorocarbon (PFC) core transferred to the gas phase and broke the hydrogel shell, releasing the dissolved oxygen. Meanwhile, US excited curcumin, causing reactive oxygen species (ROS) to be produced [22]. It was demonstrated that the oxygen‐augmented sonodynamic therapy could effectively eliminate bacteria. By spraying PO/C‐MCs onto the infected wounds of mice, the locally released curcumin can play an anti‐inflammatory role [23]. With the addition of US treatment, sonodynamic therapy can fight bacteria effectively and the released oxygen can improve the hypoxic microenvironment of the wound, accelerating wound healing [24, 25]. These results suggested that our ultrasound‐responsive PO/C‐MCs can simultaneously obtain oxygen and anti‐bacterial drug delivery, showing promising potential in promoted wound treatment.

## 2 Results and Discussion

**FIGURE 2:**
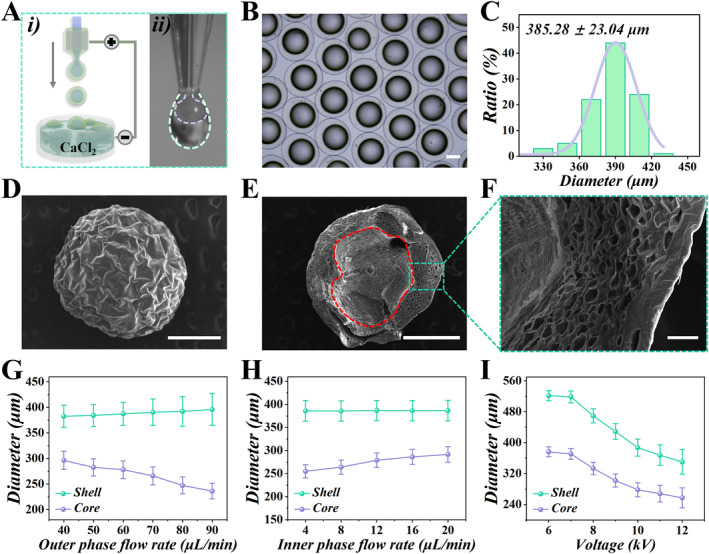
Characterization of microcapsules with a core‐shell structure. (A) Schematic (i) and high‐speed camera image (ii) of the process of microfluidic electrospray. (B) Microscope image of the core‐shell microcapsules. (C) Statistical chart of the particle size distribution of microcapsules. (D–F) SEM images of a microcapsule (D), the core‐shell structure of a microcapsule (E), and cross‐section of the alginate shell (F). The red dotted line in (E) represents the boundary between the core and the shell. (G–I) The relationship between the core and shell sizes of microcapsules and the outer phase flow rate (G), inner phase flow rate (H), and voltage (I) (*n* = 5). Scale bars represent 200 μm in (B, D, E) and 20 μm in (F).

In this study, a microfluidic device was firstly constructed to prepare core‐shell microcapsules each containing an oxygen‐enriched perfluorocarbon core and an alginate shell encapsulating Chinese medicine curcumin. This microfluidic device has two microchannels, the inner of which was perfused with oxygen‐enriched perfluorocarbon liquid, and the outer of which was perfused with curcumin‐containing sodium alginate solution. These two phases flew through the channels and were electrosprayed to form a microdroplet at a sufficient voltage (Figure 2A, Supporting Information S1: Figure S1). Calcium chloride gel bath was employed to collect the sprayed microdroplets. When collected in this solution, Ca^2+^ ions rapidly diffuse into the sodium alginate droplets, inducing ionic cross‐linking. This cross‐linking reaction causes the alginate to solidify, forming a stable shell structure [26]. Using this method of microfluidic electrospray, microcapsules with oxygen‐enriched perfluorocarbon core and hydrogel shell encapsulating curcumin could be finally obtained (Figure 2B). Parametric precision control over the microcapsule architecture, specifically core diameter and shell thickness, was attained through coordinated tuning of inner/outer phase flow rates coupled with applied voltage during microfluidic synthesis (Figure 2G–I). Finally, to obtain microcapsules with uniform particle size stably and efficiently, we tuned the outer flow rate to 60 μL/min and the inner flow rate to 12 μL/min with a voltage of 10 kV for batch production of PO/C‐MCs. Statistical analysis shows that the particle size distribution of PO/C‐MCs is between 256 and 423 μm, with an average particle size about 385.28 ± 23.04 μm (Figure 2C). To show the drug‐loading capacity and microstructure of the microcapsules, we observed these microcapsules by scanning electron microscope (SEM). It can be seen from the SEM image that the freeze‐dried microcapsules have a spherical shape with wrinkled surfaces (Figure 2D). Furthermore, the cross‐sectional scanning electron microscopy (SEM) analysis distinctly confirmed the core‐shell architecture of the microcapsules (Figure 2E,F).

**FIGURE 3:**
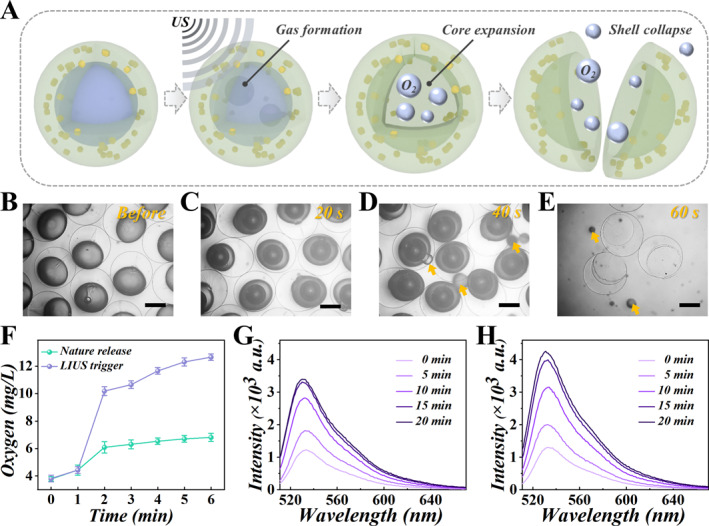
Liquid‐gas phase transition and ultrasonic responsiveness of the microcapsules. (A) Schematic illustration of ultrasound‐triggered liquid‐to‐gas phase transition in oxygen‐enriched perfluorocarbon microcapsules. (B–E) Microscopic images of oxygen‐enriched PFC‐encapsulated microcapsules before ultrasonic stimulation and after 20 s, 40 s, and 60 s of low‐intensity ultrasonic irradiation. The yellow arrows in (D, E) represent the released PFC. (F) Changes in dissolved oxygen concentration of oxygen‐enriched PFC released spontaneously and released by US (*n* = 3). (G, H) Fluorescence intensity curves of the reaction between SOSG and the heterogeneous solution of core‐shell microcapsules infused with perfluorocarbons and curcumin (P/C‐MCs) (G) and core‐shell microcapsules containing oxygen‐rich PFC and curcumin (PO/C‐MCs) (H) after US irradiation at different time intervals. Scale bars represent 200 μm in (B–E).

Perfluorocarbon (PFC) compounds exhibit chemical inertness and maintain liquid phase stability under ambient conditions (25°C, 1 atm) [27]. It has been used in clinical practice for decades due to its good biocompatibility and oxygen solubility [28]. The low boiling point of perfluorocarbon makes it susceptible to stimulated volatilization [29]. In this study, perfluorocarbon droplets were encapsulated in a hydrogel shell layer to ensure that the perfluorocarbon remains a stable liquid. In theory, exposure to external stimulation occurs to the perfluorocarbon, such as hyperthermia, the core would vaporize gradually into a larger bubble and finally break through the hydrogel shell (Figure 3A). To elucidate the ability of ultrasound‐induced liquid‐gas phase transition of perfluorocarbon core and oxygen emission from microspheres, we employed low intensity ultrasound (1.0 MHz, 1.5 W cm^−2^) to exert stimulation on perfluorocarbon cores. To better simulate physiological conditions, oxygen‐enriched perfluorocarbon microspheres were incubated in phosphate‐buffered saline (PBS) at 37°C (Figure 3B). Following 20 s of low‐intensity ultrasound (US) exposure, the partially encapsulated perfluorocarbon liquid began undergoing vaporization (Figure 3C). Under ultrasound (US) stimulation, continuous vaporization of the perfluorocarbon drove progressive expansion of the gaseous core (Figure 3D). Finally, subjected to continuous US irradiation, liquid perfluorocarbon gradually vaporized and broke through the shell of the polymer alginate (Figure 3E). Additionally, by measuring the oxygen level in PBS, we found that the US stimulation can lead to a controlled oxygen release from microcapsules (Figure 3F). These results indicated the ultrasonic responsiveness of the encapsulated perfluorocarbon, making it possible for US‐triggered inner‐cargo release.

Although the mechanism of sonodynamic therapy needs to be further investigated, oxidation induced by free radicals during acoustic dynamics has been demonstrated [30]. The cavitation effect of US excitation can lead to acoustic‐thermal reactions, such as acoustic sensitization through acoustic energy stimulation to peak energy levels, and when de‐excitation of the acoustic sensitizer induces collisional energy transfer to adjacent molecular oxygen, followed by the generation of ROS, especially ^1^O_2_ that can damage cells through peroxidation [31, 32]. To assess in vitro ^1^O_2_ To evaluate ^1^O_2_ production in vitro, we employed Singlet Oxygen Sensor Green (SOSG), a fluorescent probe exhibiting significantly enhanced emission upon reaction with ^1^O_2_ [33]. To explore the sonosensitivity of the microcapsules and the auxiliary role of external supplied oxygen, we compared the changes of SOSG in the P/C‐MCs (microcapsules with untreated PFC core and curcumin‐encapsulated shell) group and PO/C‐MCs group. SOSG fluorescence emission exhibited progressive intensification proportional to ultrasound exposure duration, signifying time‐dependent singlet oxygen accumulation (Figure 3G), suggesting that ^1^O_2_ could be produced by the P/C‐MCs system under low intensity US irradiation. Notably, we noted that the production of ^1^O_2_ was further increased in the presence of PO/C‐MCs (Figure 3H, Supporting Information S1: Figure S2), which was due to the external supplied oxygen from the oxygen‐enriched perfluorocarbon core under US irradiation.

**FIGURE 4:**
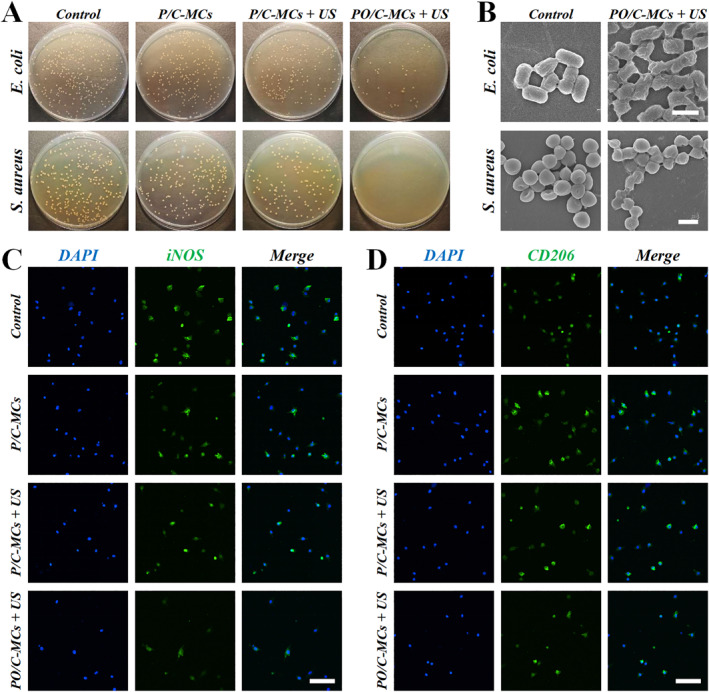
The sonodynamic antibacterial and anti‐inflammatory effect of oxygen and curcumin‐loaded microcapsules. (A) Colony formation of *E. coli* and *S. aureus* after therapy with P/C‐MCs, P/C‐MCs + US, and PO/C‐MCs + US. (B) Representative scanning electron microscopy (SEM) images revealing morphological alterations in *E. coli* and *S. aureus* following combined PO/C‐MCs and ultrasound (US) treatment. (C, D) Characterizations of macrophage polarization by immunofluorescence staining of DAPI (blue), iNOS (green), and CD206 (green). Scale bars are 1 μm in (B) and 100 μm in (C, D).

To demonstrate the value of PO/C‐MCs in the treatment of infected wounds, we investigated the inhibitory effects of ultrasound‐excited PO/C‐MCs on *Staphylococcus aureus* (*S. aureus*) and *Escherichia coli* (*E. coli*). The bacteria were randomly divided into four groups, that is, control group, P/C‐MCs group, P/C‐MCs + US group and PO/C‐MCs + US group. The control group received no therapeutic intervention, bacteria in the P/C‐MCs group and P/C‐MCs + US group were co‐incubated with P/C‐MCs, while US stimulation (1.0 MHz center frequency and 1.5 W cm^−2^ intensity power) was included in the P/C‐MCs + US group. PO/C‐MCs and US stimulation was applied to the PO/C‐MCs + US group. Through the colony formation experiment, it can be observed that the number of colonies of *S. aureus* and *E. coli* was significantly reduced in the latter three groups, especially in the PO/C‐MCs + US group (Figure 4A, Supporting Information S1: Figure S3). SEM was further used to observe the morphologies of bacteria before and after treatment (Figure 4B). The *S. aureus* and *E. coli* treated by PO/C‐MCs and US stimulation showed obvious membrane destruction, suggesting that the PO/C‐MCs system has good antimicrobial properties, which may be useful for treating anti‐bacterial treatment.

Then, we explored the anti‐inflammatory effect of the PO/C‐MCs system. RAW 264.7 macrophages were randomly assigned to four experimental groups: (1) untreated control, (2) P/C‐MCs alone, (3) P/C‐MCs with ultrasound (US) stimulation, and (4) PO/C‐MCs with US stimulation. The intervention measures are as described above. Compared with the control group, macrophages in the P/C‐MCs group showed significant repolarization from M1 to M2, indicating the anti‐inflammatory efficacy of the Chinese herb curcumin (Figure 4C,D, Supporting Information S1: Figure S4). Notably, with the application of US, macrophages not only showed polarization toward the anti‐inflammatory type, but also decreased in number, especially in the PO/C‐MCs + US group. These results might be due to the sonodynamic effect between curcumin and US.

**FIGURE 5:**
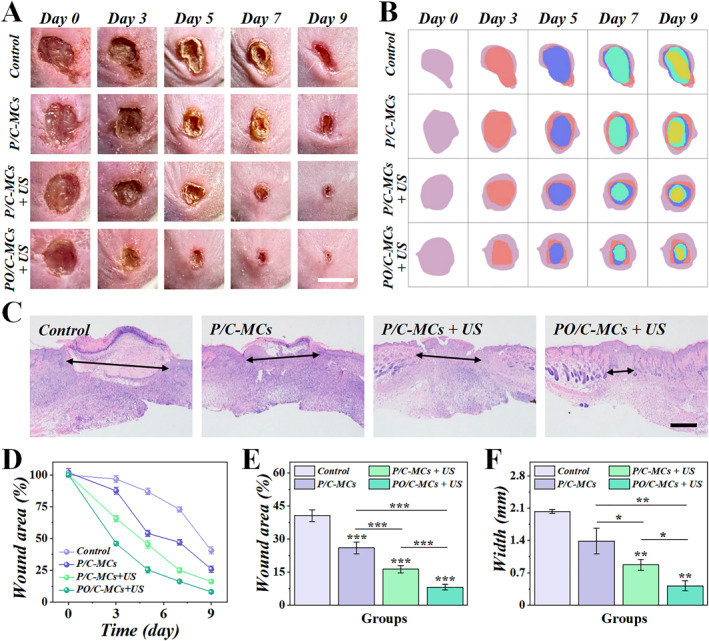
The PO/C‐MCs accelerated the wound healing process of the bacterial infected wounds. (A) Serial macroscopic images documenting wound morphological progression under distinct treatments at days 0, 3, 5, 7, and 9 post‐interventions. (B) Schemes of the wound area variation on day 0, 3, 5, 7, and 9. (C) HE images of the skin tissues from different groups. (D) Statistical analyses of wound area of different groups. (E) The wound area comparison between different groups on day 9. (F) Statistical analyses of the wound width of different groups according to HE images. Scale bars are 8 mm in (A), 500 μm in (C). (*n* = 5).

Wound healing progresses through a dynamic cascade of physiological stages—a process significantly compromised in infected wounds. Bacterial colonization impairs cellular nutrient and oxygen uptake capacity, thereby inhibiting tissue regeneration and angiogenesis [34]. Building upon the demonstrated oxygenation capacity and antimicrobial efficacy from in vitro studies, we assessed the in vivo therapeutic potential of PO/C‐MCs with ultrasound (US) activation for treating *Staphylococcus aureus*‐infected wounds. Before animal experiments, we verified the biocompatibility of the microcapsules using 3T3 cells (Supporting Information S1: Figure S5). Full‐thickness dermal wounds (8 mm diameter) were generated on mouse dorsa using biopsy punches, with subsequent topical application of *S. aureus* suspension (1 × 10^8^ CFU/mL in PBS) to establish infected wounds (Figure 5A). Mice were randomly assigned to four treatment groups: (1) untreated control, (2) P/C‐MCs alone, (3) P/C‐MCs with ultrasound (US) stimulation, and (4) PO/C‐MCs with US stimulation. The wound healing process was photographed on days 0, 3, 5, 7, and 9 in all groups, and there was a gradual decrease in wound size in all groups over time (Figure 5B,D). The infected wounds in the control group healed the slowest, and even pus formation could be observed on the surface. However, the infected wounds treated with PO/C‐MCs + US had the fastest healing process. In addition, we further evaluated the tissue regeneration by Hematoxylin‐Eosin (HE) staining. The epithelial regeneration was the least in the control group, while it was more in the PO/C‐MCs + US group (Figure 5C). Further evidence that PO/C‐MCs + US significantly shortened the healing time of infected wounds was demonstrated by quantitative analyses of wound closure rate and wound width (Figure 5E,F). Additionally, HE staining of the main organs verified the biocompatibility of our PO/C‐MCs system (Supporting Information S1: Figure S6). The changes in wound morphology and healing time in different groups indicated that acoustic antimicrobial therapy with curcumin plus ultrasound irradiation plays an important role in promoting wound healing.

**FIGURE 6:**
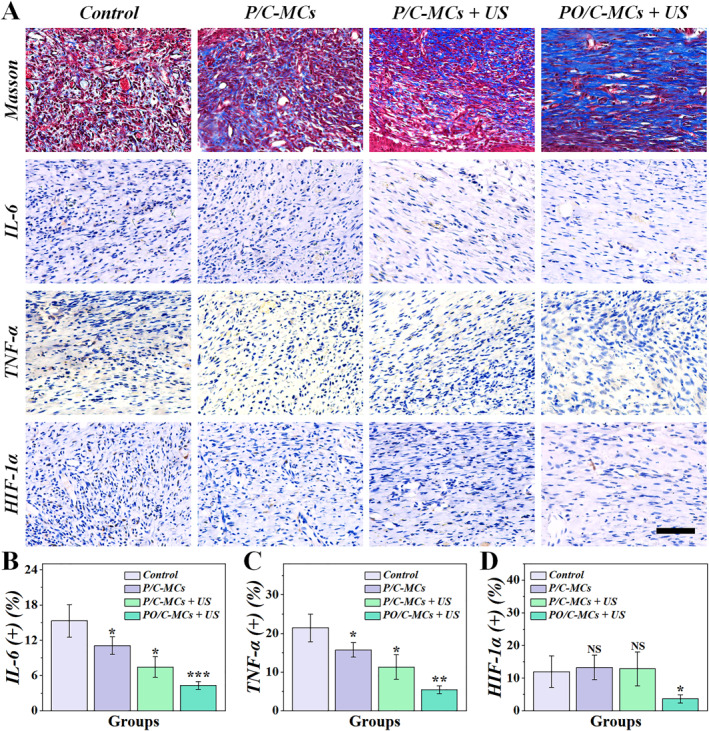
Immunohistochemical analysis of the wound repair mechanism. (A) Immunohistochemical staining of Masson, IL‐6, TNF‐α, and HIF‐1α. (B–D) Quantitative analyses of IL‐6 (B), TNF‐α (C), and HIF‐1α (D). Scale bar is 50 μm. (*n* = 5).

To further investigate the inflammatory response at the wound site, we conducted Masson, interleukin (IL‐6), tumor necrosis factor‐α (TNF‐α), and hypoxia‐inducible factor 1α (HIF‐1α) staining. The highest amount of collagen deposition was observed in the PO/C‐MCs + US group compared to the control, P/C‐MCs , and P/C‐MCs + US groups (Figure 6A). Quantitative results also showed that the area of collagen deposition was the largest in the PO/C‐MCs + US group (Supporting Information S1: Figure S7). Figure 6A illustrates that IL‐6 levels were markedly increased in the control group, suggesting a severe inflammatory response. Compared with the control groups, the expression levels were significantly lower in the PO/C‐MCs + US group (Figure 6B), which may be due to the anti‐inflammatory properties of curcumin itself, as well as the antimicrobial effect of curcumin in combination with US. Similar changing trends can be observed in the TNF‐α staining results (Figure 6A,C).

While transient hypoxia in acute wounds facilitates angiogenesis during early healing, persistent hypoxia in chronic wounds induces vascular regression and necrotic tissue formation [35]. Hypoxic status within granulation tissue was quantified via HIF‐1α immunohistochemical staining, where nuclear or cytoplasmic brown granules indicated positive immunoreactivity. Comparative analysis revealed significantly attenuated HIF‐1α expression in the PO/C‐MCs + US cohort relative to all other treatment groups (*p* < 0.01) (Figure 6A,D). These results suggested that our Chinese medicine and oxygen integrated microcapsules accompanied by the irradiation of US can improve the hypoxia of the traumatized tissues.

## 3 Conclusion

In this study, we proposed a novel microcapsule combining Chinese medicine anti‐inflammatory treatment and sonodynamic antibacterial therapy. Based on the anti‐inflammatory and sonosensitivity of curcumin, we designed a core‐shell structure microcapsule with an alginate hydrogel shell carrying the curcumin, and a perfluorocarbon core delivering oxygen. The encapsulated curcumin can release from the shell to exert anti‐inflammatory effect, and notably, with the stimulation of external US, curcumin can be excited and realize sonodynamic therapy. It is interesting that along with the US‐responsiveness of the perfluorocarbon core, the controllable oxygen release can contribute to the ROS generation during the sonodynamic process, significantly enhancing therapeutic efficacy. By treating the infected wounds on mice, it was verified that our PO/C‐MCs treating platform can accelerate wound closure, collagen deposition and convert hypoxia conditions. Therefore, our US‐responsive oxygen and curcumin microcapsules provide a new platform for Chinese medicine application and an effective strategy for enhancing sonodynamic therapy.

## 4 | Experimental Section

### 4.1 Production of Core‐Shell Microcapsules

The precursor of shell containing 2.5 wt.% sodium alginate (low viscosity) and 0.02 M curcumin. Oxygen‐enriched perfluorocarbon liquid was prepared by continuously introducing oxygen into the perfluorocarbon liquid for 15 min. The collecting bath contained 1.5 wt.% calcium chloride. Two syringe pumps were applied to push the two‐phase liquid at a constant speed. To establish parametric relationships between microcapsule dimensions (core diameter/shell thickness) and flow conditions, we systematically modulated inner/outer phase flow rates during microfluidic synthesis. High voltage director equipment was connected between the orifice of microchannels and collecting bath. Different voltages were set to evaluate the relationship between the core and shell sizes of microcapsules and voltage.

### 4.2 US Responsiveness of the Perfluorocarbon Core

Microcapsules for subsequent experiments were fabricated using 60 μL/min outer flow rate, 12 μL/min inner flow rate, and 10 kV voltage. The microcapsules were placed in PBS at 37°C, accompanied by US irradiation (1.0 MHz, 1.5 W cm^−2^). The morphologies of the microcapsules were recorded using a stereomicroscope after 0, 20, 40, and 60 s US stimulation. A dissolved oxygen detector was applied to measure the oxygen level in the PBS.

### 4.3 Sonosensitivity of Curcumin

Microcapsules with perfluorocarbon core and oxygen‐enriched perfluorocarbon core were subjected to irradiation with ultrasound (1.0 MHz, 1.5 W cm^−2^) for 0, 5, 10, 15, and 20 min. The selection of ultrasound conditions accords with our previous study [32]. At various time intervals, 100 μL of supernatant was separated and combined with SOSG solution (20 mg/L). Then, the fluorescence intensities of different groups were measured using a microplate reader (excitation: 504 nm, emission: 522 nm).

### 4.4 Anti‐Bacterial Analyses

Bacterial suspensions of *S. aureus* and *E. coli* were allocated to four experimental cohorts: (1) Untreated controls; (2) P/C‐MC exposure alone; (3) P/C‐MCs with ultrasound (US) stimulation; and (4) PO/C‐MCs with US stimulation. The bacteria were treated by different microcapsules with or without US irradiation. Then, the bacteria were incubated at 37°C for 8 h, followed by the SEM observation and spread plate operation.

### 4.5 Animal Experiments

Twenty Balb/c were randomly assigned to the control group, P/C‐MCs group, P/C‐MCs + US group, and PO/C‐MCs + US group after establishment of infected wounds. In the control group, the mice were left untreated. In the P/C‐MC group, microcapsules were placed on the wound. In the P/C‐MCs + US group, and PO/C‐MCs + US group, different microcapsules were placed on the wound followed by treatment with ultrasonic waves at 1.0 MHz and 1.5 W cm^−2^ for 2 min. The wounds were recorded on days 0, 3, 5, 7, and 9. The wound closure area and wound width were measured using the ImageJ software. The wound healing rate was calculated by comparing the wound area on the 3rd, 5th, 7th, and 9th days to that on the 0th day, with the former divided by the latter. The wound width was measured on the HE images of the wound surfaces in each group. On day 9, mice were sacrificed and the skin samples were taken for further analyses. The Animal Ethics Committee of Drum Tower Hospital, which is affiliated with the Medical School of Nanjing University (2023AE01078), established the guidelines for all animal experiments.

### 4.6 Statistical Analyses

All statistical results were analyzed by Origin 2019b software. The data are presented as mean ± standard deviation. Comparative analyses were conducted using the two‐sided Student's t test. *p* < 0.05 was defined as statistically significant. NS: no statistical significance, *: *p* < 0.05, **: *p* < 0.01, and ***: *p* < 0.001.

## Author Contributions

**Baojie Wen:** conducted the research, wrote the manuscript and drew all the figures. **Danqing Huang:** assisted with manuscript writing – review and editing. **Chuanhui Song:** assisted with manuscript writing – review and editing. **Yi Chen** assisted with manuscript writing – review and editing. **Yuanjin Zhao:** conceived the idea, funding acquisition, supervision.

## Ethics Statement

The animal experiments have received approval from the Animal Investigation Ethics Committee of Drum Tower Hospital (2023AE01078).

## Conflicts of Interest

The authors declare no conflicts of interest.

## Supporting information


Supporting Information S1

## Data Availability

The data supporting the findings of this study can be obtained from the corresponding author upon reasonable request.
