# Supplementary material for: Ultrasound‐Responsive Microcapsules Delivering Oxygen and Traditional Chinese Medicine for Wound Healing
Source: Smart Med. 2025 Nov 16;4(4):e70021. doi: 10.1002/smmd.70021 (PMC12622483; doi:10.1002/smmd.70021)
Supplement: Supplementary file 1 — Supporting Information S1 [file SMMD-4-e70021-s001.docx]

Supporting Information

Ultrasound-responsive microcapsules delivering oxygen and traditional Chinese medicine for wound healing

Baojie Wen, Danqing Huang, Chuanhui Song, Yi Chen, Yuanjin Zhao*

**Supporting Figures**


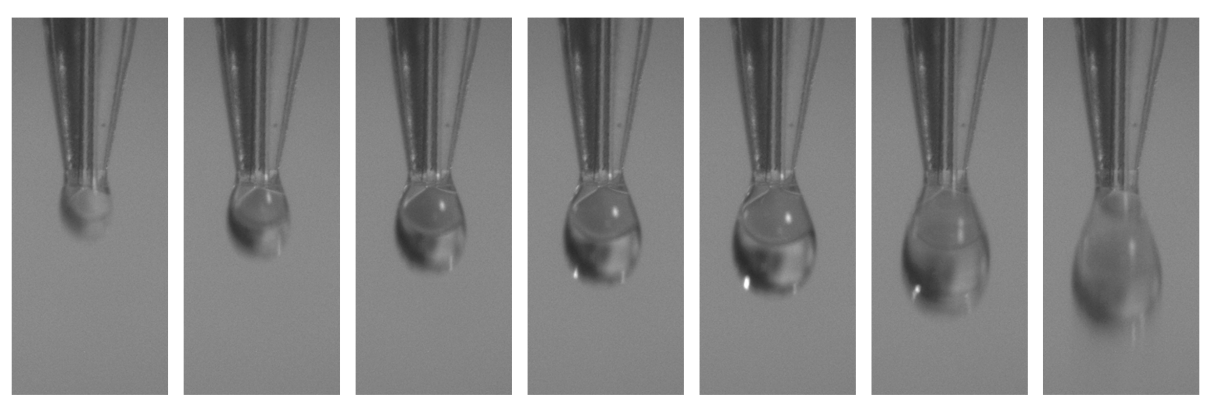


**Figure S1.** High-speed camera photographs of formation of core-shell microparticles.


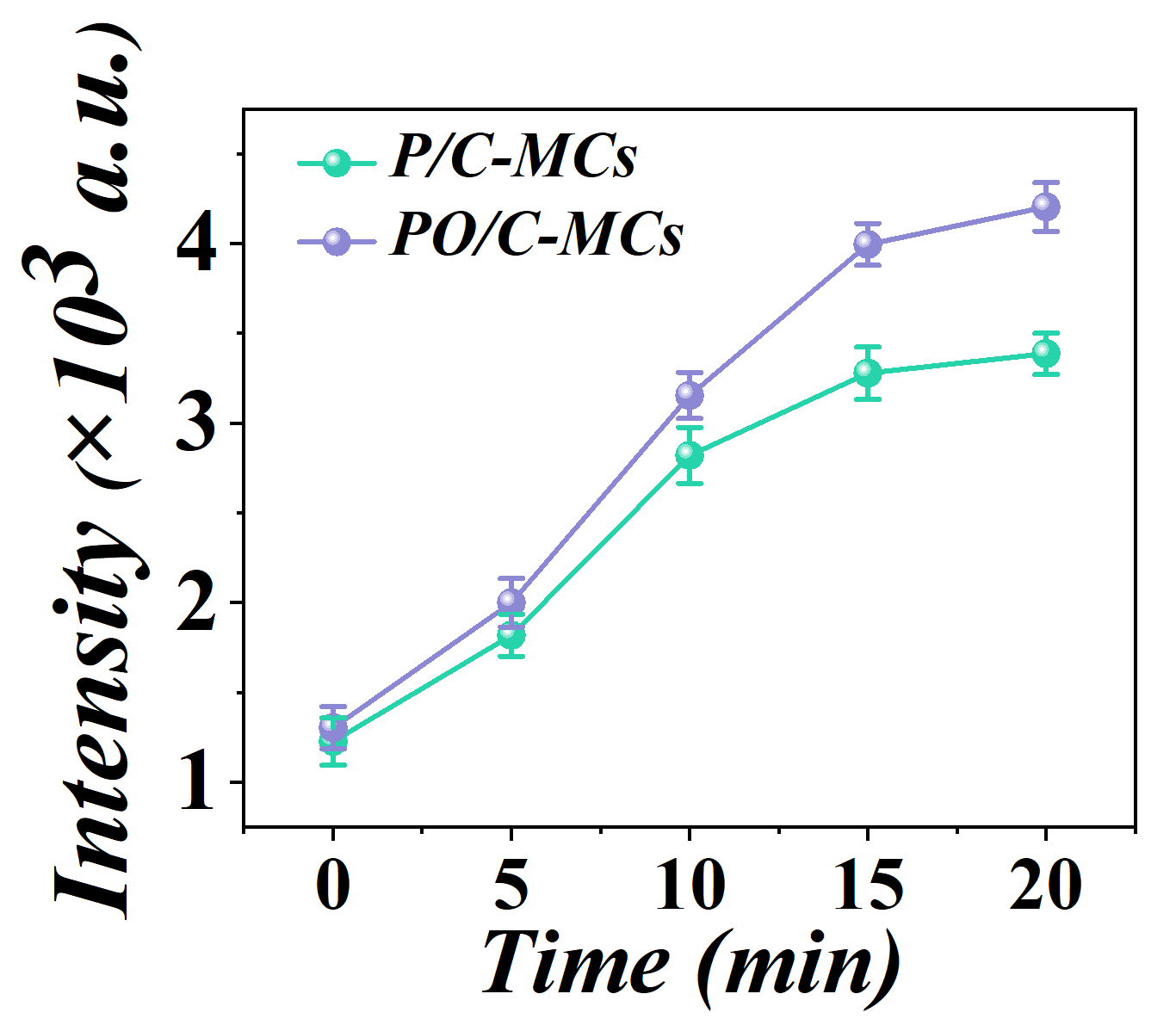


**Figure S2.** Absorbance peaks of different groups at different time intervals.


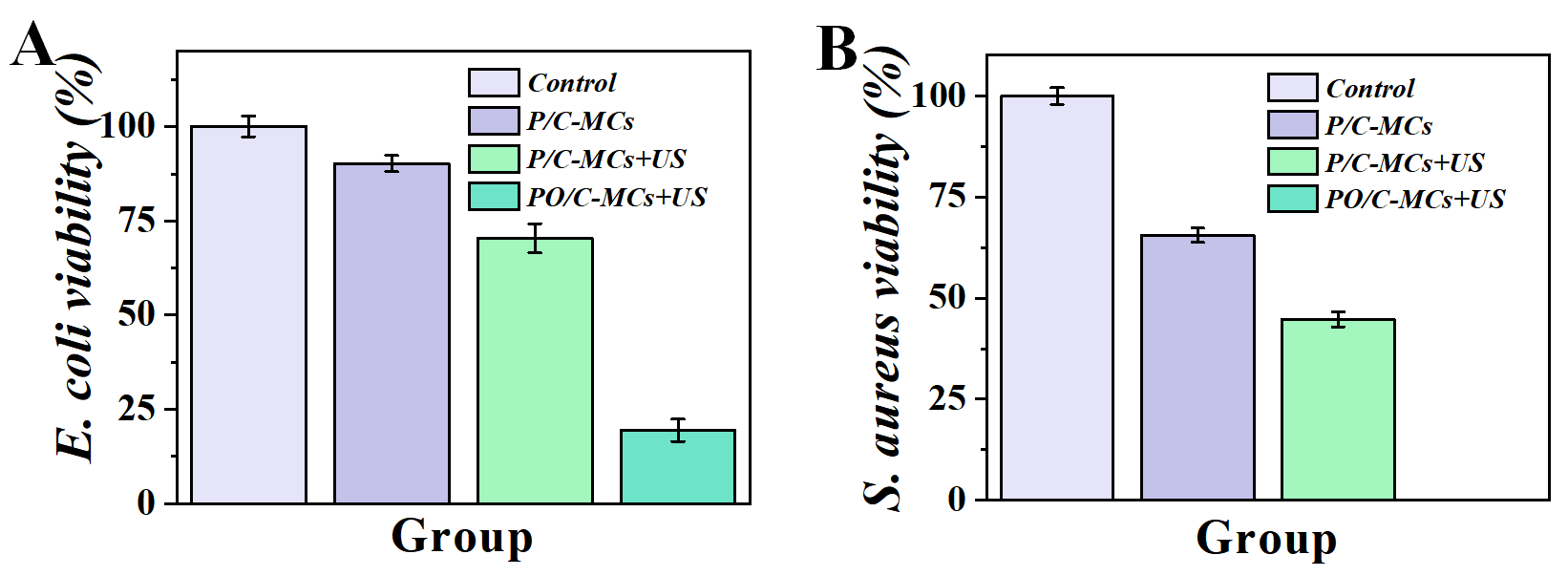


**Figure S3.** Quantitative analyses of the anti-bacteria effect of PO/C-MCs + US on *E. coli* (A) and *S. aureus* (B).


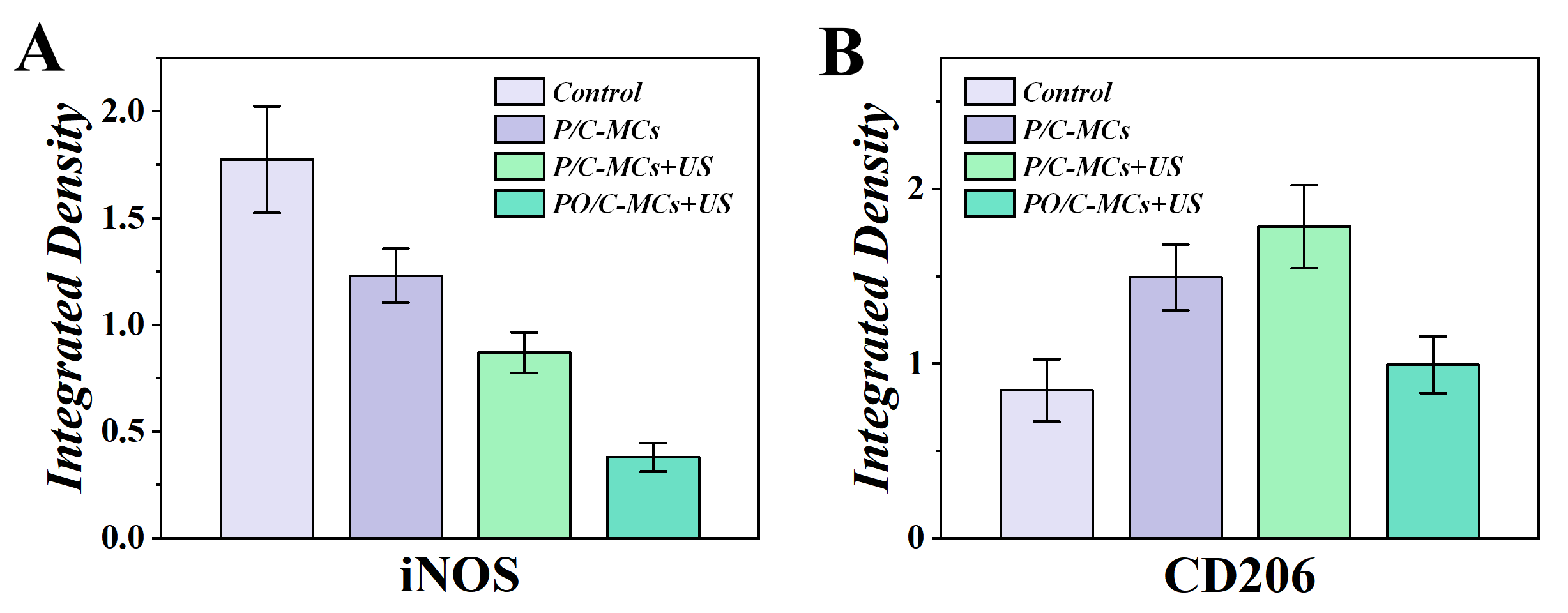


**Figure S4.** Quantitative analyses of the macrophage re-polarization. (n = 3)


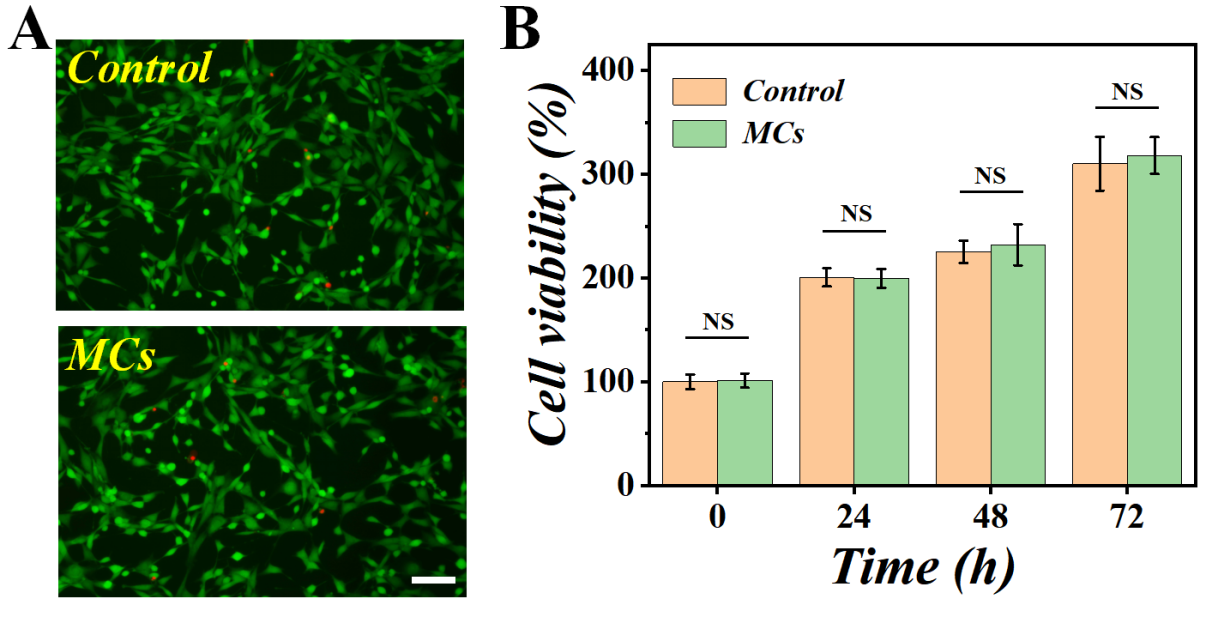


**Figure S5.** Biocompatibility study of the microcapsules on 3T3 cells. (A) Live/dead staining images of 3T3 cells cultured in blank medium and co-cultured with microcapsules for 48 h. Scale bar is 100 μm. (B) CCK-8 results of 3T3 cells at different time periods.


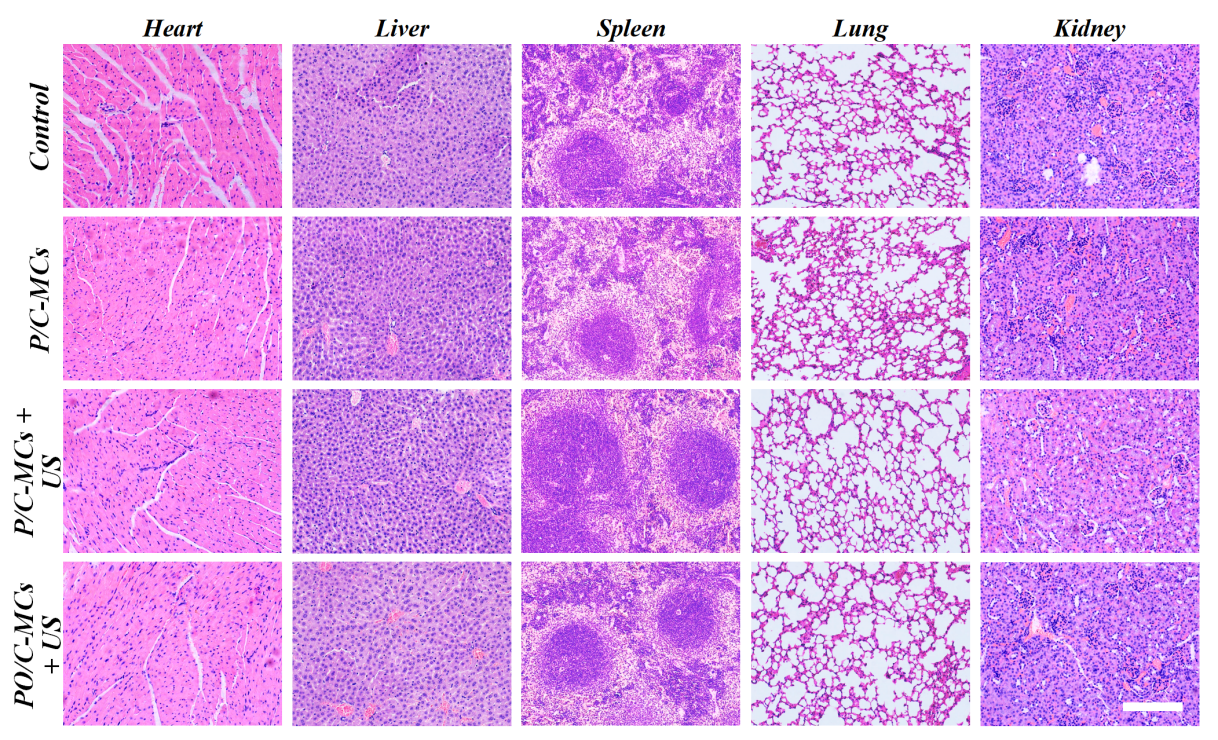


**Figure S6.** Hematoxylin and eosin staining of principal organs in each experimental group of mice. The scale bar measures 200 micrometers.


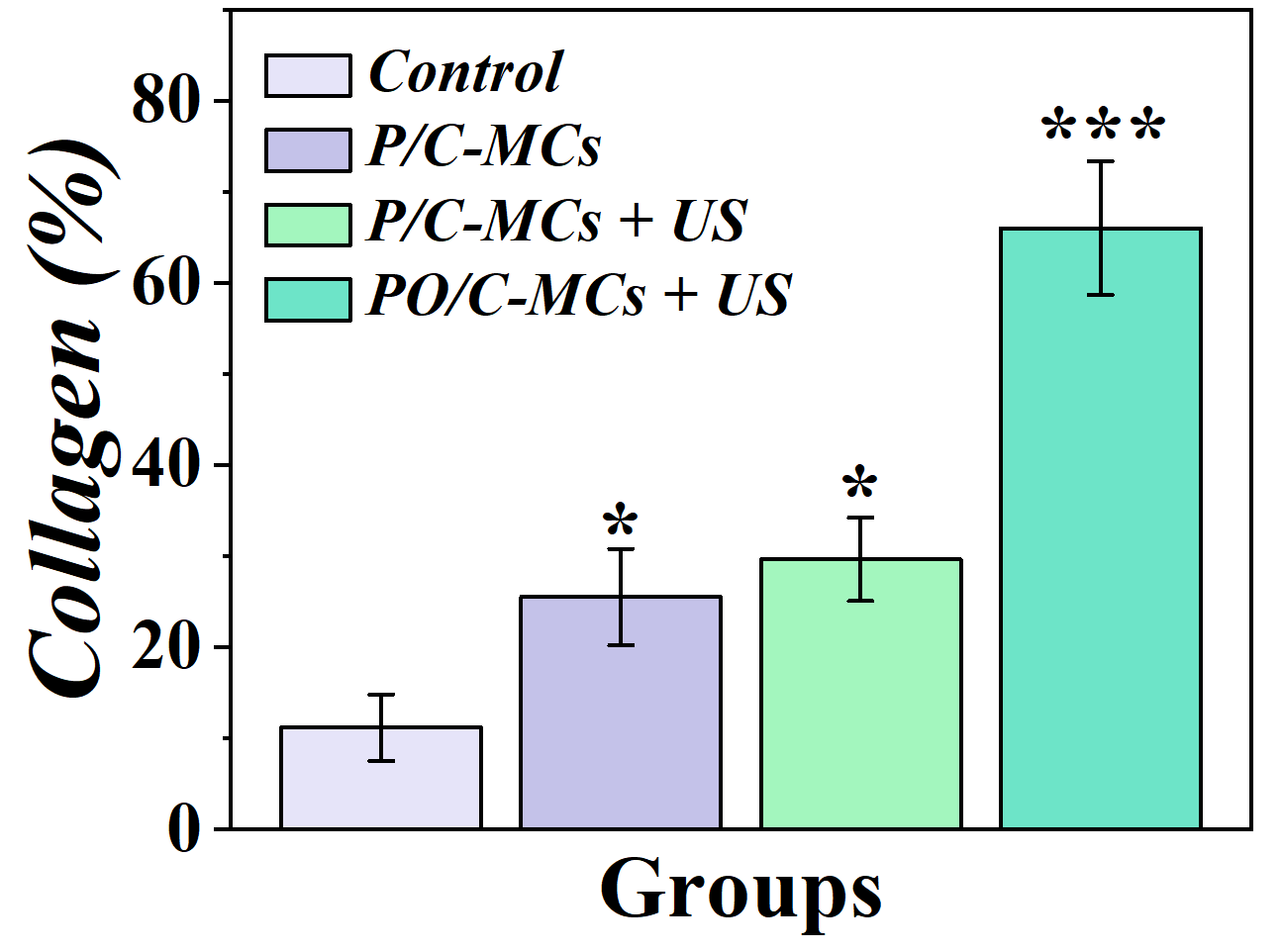


**Figure S7.** Quantitative analysis of collagen deposition from different groups.
